# Discovering Condition-Specific Gene Co-Expression Patterns Using Gaussian Mixture Models: A Cancer Case Study

**DOI:** 10.1038/s41598-017-09094-4

**Published:** 2017-08-17

**Authors:** Stephen P. Ficklin, Leland J. Dunwoodie, William L. Poehlman, Christopher Watson, Kimberly E. Roche, F. Alex Feltus

**Affiliations:** 1 Department of Horticulture, Washington State University, Pullman, WA 99164 USA; 2 Department of Genetics & Biochemistry, Clemson University, Clemson, SC 29631 USA; 3 Molecular Plant Sciences Program, Washington State University, Pullman, WA 99164 USA

## Abstract

A gene co-expression network (GCN) describes associations between genes and points to genetic coordination of biochemical pathways. However, genetic correlations in a GCN are only detectable if they are present in the sampled conditions. With the increasing quantity of gene expression samples available in public repositories, there is greater potential for discovery of genetic correlations from a variety of biologically interesting conditions. However, even if gene correlations are present, their discovery can be masked by noise. Noise is introduced from natural variation (intrinsic and extrinsic), systematic variation (caused by sample measurement protocols and instruments), and algorithmic and statistical variation created by selection of data processing tools. A variety of published studies, approaches and methods attempt to address each of these contributions of variation to reduce noise. Here we describe an approach using Gaussian Mixture Models (GMMs) to address natural extrinsic (condition-specific) variation during network construction from mixed input conditions. To demonstrate utility, we build and analyze a condition-annotated GCN from a compendium of 2,016 mixed gene expression data sets from five tumor subtypes obtained from The Cancer Genome Atlas. Our results show that GMMs help discover tumor subtype specific gene co-expression patterns (modules) that are significantly enriched for clinical attributes.

## Introduction

Gene co-expression networks GCN (also known as relevance networks^1^) are mathematical graphs that are increasingly used to model the co-expression relationships between genes. Within a GCN, genes (or gene products) serve as nodes and edges exist between two genes when their expression profiles are correlated across a set of expression-measurement samples (e.g. microarray or RNA-seq). GCNs typically exhibit common graph theory principles such as scale-free, modular, and hierarchical behavior^2^. Highly connected groups of genes are often referred to as modules or clusters, and it has been shown that their member genes tend to be involved in similar biological functions^3^. Thus, the principle of guilt-by-association^4^ is a powerful method to predict novel contributor genes from GCNs. A form of GCN was first reported by Eisen *et al*.^5^ and GCNs have since been used for a variety of species-specific analyses, including cancer studies^6–9^. Additionally, GCN’s can be used in a systems genetics^10^ approach by integrating genetics data (e.g. Quantitative Trait Loci (QTLs)^11, 12^ or SNPs from Genome Wide Association Studies (GWAS)^13–15^) to associate modules to traits of interest^16–19^.

Despite the increased use of GCNs, the accuracy of the modules within a GCN are negatively affected by “noise”. The sources of noise that can affect a GCN are diverse and are introduced at various stages of the network construction workflow. First, gene expression itself is known to be noisy due to intrinsic natural variation amongst cells even in the same environment^20–22^. Second, noise can be introduced due to natural extrinsic variation such as changes in environmental conditions, or differences due to genotype, developmental stage, tissue type, etc. Third, systematic noise is introduced from techniques used to measure gene expression^23^. Individual labs, sample preparation protocols, and instruments, all generate bias within the collected dataset. This is a known problem in both microarray and RNA-seq data collections. Fourth, software used to convert output from the instrument into gene expression levels often attempt to correct for systematic bias. For microarrays, some methods include Robust Multichip Average (RMA)^24^, Affymetrix Microarray Suite MAS5^25^, and Factor Analysis for Robust Microarray Summarization (FARMS)^26^ to name a few. For RNA-seq, some examples include Reads Per Kilobase per Million mapped reads (RPKM)^27^, Remove Unwanted Variation (RUV)^28^, and approaches provided by software for differential gene expression analysis such as DESeq^29^. However, these methods can introduce bias and studies comparing the efficacy of these various methods are available^30–32^. Fifth, Pearson and Spearman correlation and Mutual Information (MI) are popular methods for identifying co-expression among genes. However, due to statistical bias, each method can yield different networks from the same input data. Srihari and Ragan sought to resolve these differences by accounting for multiple correlation methods in their final network construction scheme^7^ and Kumari *et al*. provide recommendation as to which methods work best at identifying co-expressed genes in molecular pathways versus regulation^33^. Song *et al*. provide a comparison of popular correlation methods^34^. Lindlöf and Lubovac showed no significant improvement in the quality of networks constructed using Pearson versus MI^35^, but, Song *et al*. showed that “MI is often inferior to correlation based approaches in terms of elucidating gene pairwise relationships and identifying co-expression modules”. A variety of software tools exists for GCN construction with each employing different approaches for identifying co-expression. These include tools such as, WGCNA^36^, CLR^37^, MRNET^38^, RMTGeneNet^39^, petal^40^ and FastGCN^41^. WGCNA is the most popular network construction tool in terms of citations.

Some studies use hundreds to thousands of samples to create a GCN^42–45^. The use of hundreds of samples for GCN construction is increasingly possible when samples are obtained and combined from repositories including NCBI’s Short Read Archive (SRA)^46^, NCBI’s Gene Expression Omnibus (GEO)^47^, the European Bioinformatics Institute’s ArrayExpress database^48^, and The Cancer Genome Atlas^49^ to name a few. However, the number of samples collected and the experimental designs used to create those samples will produce a dataset with increased effects from both extrinsic and systematic noise. Freytag *et al*. report that systematic noise can be accounted for using their RUV^23^ method, but, the effect of extrinsic noise, as demonstrated by early work from Reverter and Chen^50^, remains a problem. Some effort has been made to address extrinsic noise by subdividing large sets of samples into groups prior to network construction either automatically^51^ or manually^52^, and building separate networks for each group.

To further address the problem of extrinsic noise, we report here on the use of Gaussian Mixture Models (GMMs) to discover sample modes prior to each pairwise gene correlation test. To demonstrate the benefit for GMMs, we show that commonly used correlation methods such as Spearman or Pearson are not appropriate in the presence of extrinsic noise. For example, a gene pair may have two modes of expression (a high mode and low mode) which appear as two separated clusters on a scatterplot. If the distance between those “modes” is large enough, Pearson and Spearman will report correlation which may result in a co-modality edge being introduced into the co-expression network. GMMs, however, can be used to identify the “modes” within a pairwise gene comparison (see the Methods section for an explanation of GMMs).

Use of mixture models with gene expression data is not new. Recently, a Poisson mixture model has been applied to pre-clustering of the input Gene Expression Matrix (GEM) (an *n* x *m* data set with *n* rows of transcripts and *m* columns of samples) into mixture components of genes with similar expression patterns^53^. A novel visualization using these clusters was proposed that shows the proportion of reads attributed to each condition within the clusters identified. Thus, clusters of genes with high or low association with specific traits can be visualized without construction of a network. In contrast, this work applies GMMS during network construction, prior to each pair-wise correlation calculation to identify the modes at the gene pairwise comparison.

Our hypothesis, and the motivation behind this work, is that the presence of modes of a pairwise gene comparison can be representative of condition-specific gene co-expression and these modes can be identified using GMMs. While challenges related to intrinsic, systematic and statistical noise still exist, the focus of this work is to address extrinsic noise that is exacerbated in large collections of mixed condition input samples. The GMM approach could be incorporated into any existing tool, but in this study we add support for GMMs into the open-source Knowledge Independent Network Construction (KINC) software package. KINC is freely available at http://www.github.com/SystemsGenetics/KINC and is the successor of the RMTGeneNet package^54^.

## Results

### The Effects of Extrinsic Noise on Pairwise Expression Comparison

**Figure 1:**
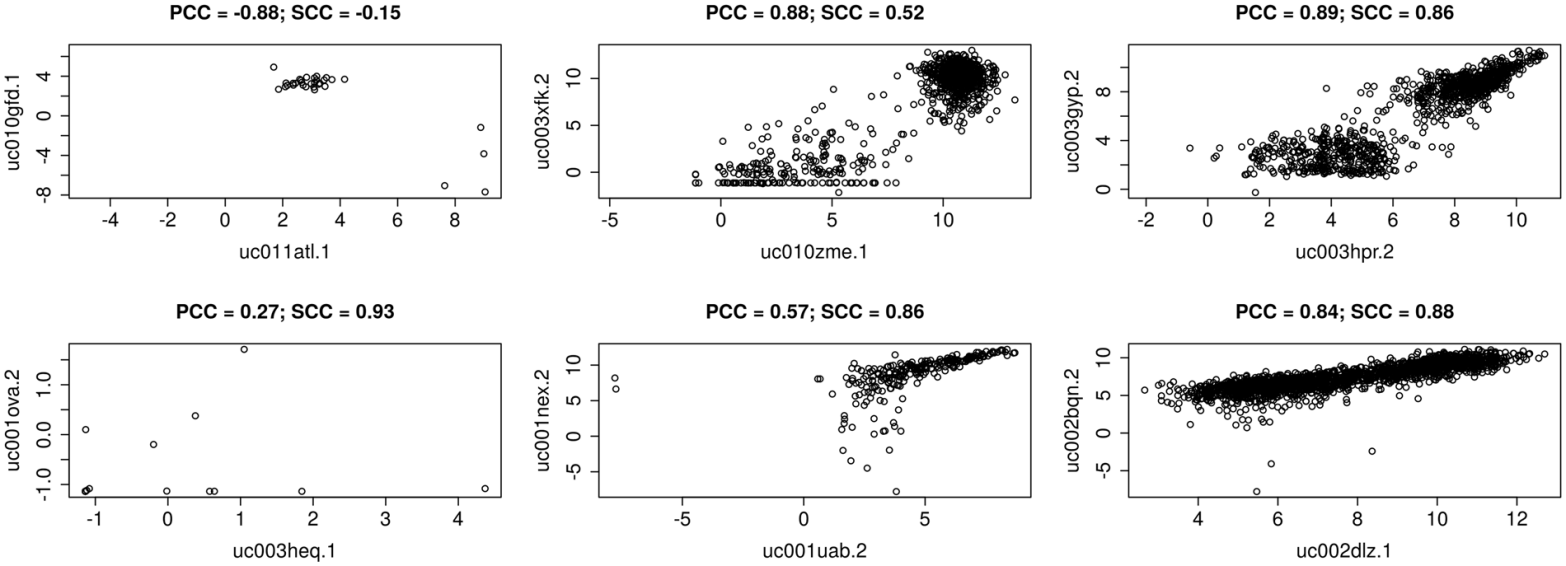
High, Medium, and Low Differences in Gene Expression Dependency. These scatterplots provide examples of high, medium and low differences in correlation between the Spearman and Pearson correlation methods. The x and y-axes represent log2 transformed gene expression levels for each gene respectively. The two plots on the left (top and bottom) represent pairwise correlation between transcripts with high differences between correlation where either Pearson correlation coefficient (PCC) is high and Spearman correlation coefficient (SCC) is low or vice versa. There are fewer samples when compared to other plots because of missing values. The middle two plots represent high correlation in one method and mid-range in the other. The right two plots are examples where both PCC and SCC are high. The title of each scatterplot indicates the PCC and SCC values for each comparison.

As mentioned previously, distinct modes of expression can be observed in some gene pairwise expression comparisons. If these modes are properly separated they can lead to the introduction of false edges due to co-modality rather than co-expression. The source of these erroneous edges become apparent when observed within scatterplots. Figure 1 provides various examples where patterns of modality yield various combinations of high, medium and low Pearson correlation coefficients (PCC) and Spearman correlation coefficients (SCC). The examples shown were selected at random from high, medium, and low ranges of difference between PCC and SCC. In the top-left panel, outliers are the cause of high negative PCC. In the top middle plot, two modes of high density points yield a high PCC and moderate SCC. If this comparison were used in a PCC-based network an erroneous edge is introduced. However, each mode, when considered separately, appears uncorrelated. Again, in the top right plot there are two distinct modes. Both Pearson and Spearman result in high correlation, although the lower expressed mode does not appear correlated on its own. The lower right plot appears linear but a thinning in the middle may indicate two different modes of expression. Again, we hypothesis that the distinct modes evident in these plots may be due to condition-specific expression.

### The GMM Gene Co-Expression Network

**Figure 2:**
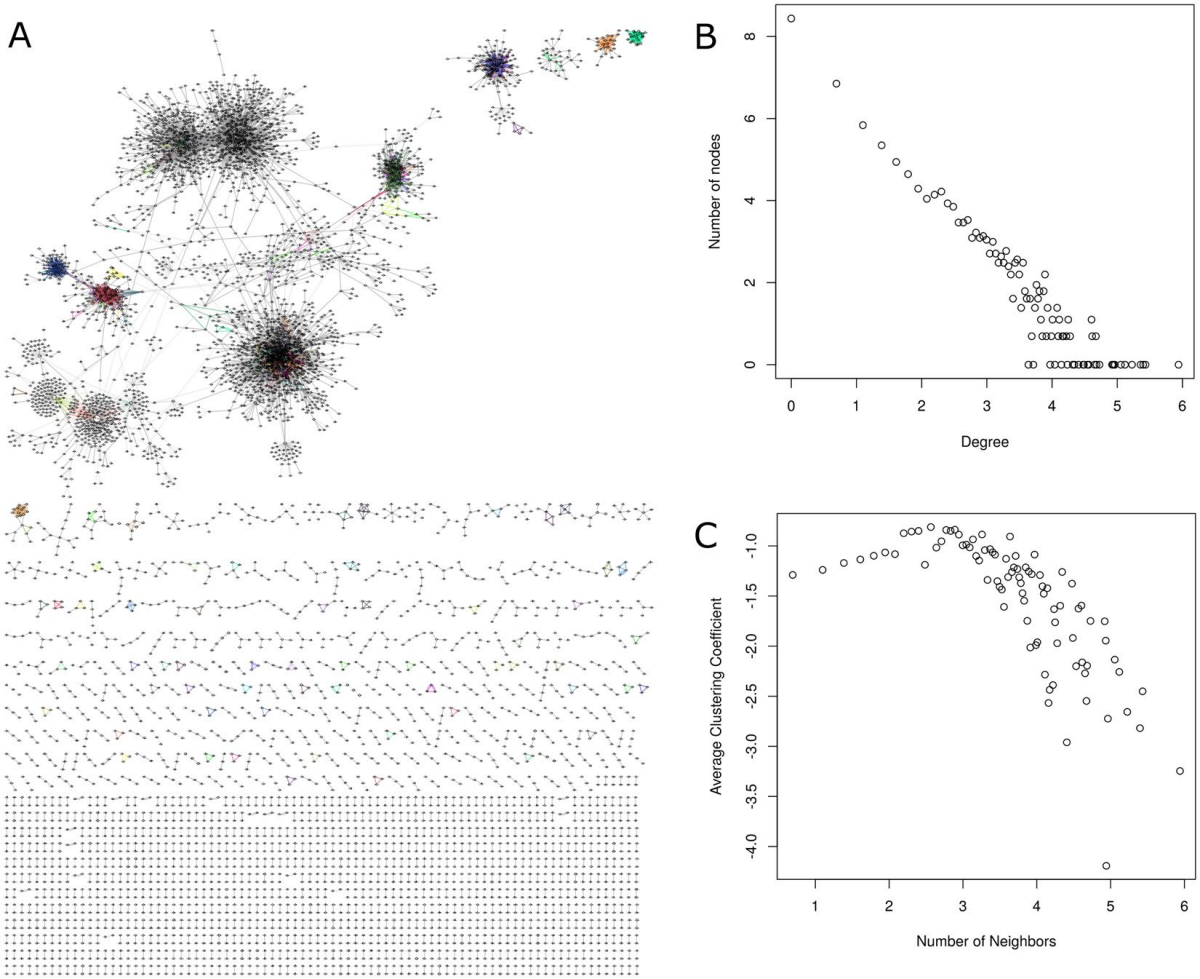
The Tumor GMM Gene Co-expression Network. (**A**) The graph representation of the network. Points represent nodes (i.e. transcripts) and edges represent co-expression of transcripts. Modules are identified using the link communities method and uniquely colored. Not all nodes were circumscribed into a module. (**B**) The node degree distribution plot demonstrating scale-free behavior of the network. (**C**) The average clustering coefficient plot demonstrating a hierarchical network.

To isolate modes in pairwise comparisons, GMM was employed during network construction. To construct the GMM-based network, a total of 2016 tumor RNA-seq datasets from The Cancer Genome Atlas (TCGA) were obtained that included all normalized isoform datasets involving lower grade glioma (LGG), thyroid cancer (THCA), glioblastoma (GBM), ovarian cancer (OV), and bladder cancer (BLCA). The global gene expression profiles of these datasets are shown in the heat map of Supplemental Figure S1. A significance threshold at a Spearman correlation of 0.8601, using Random Matrix Theory^55^, was identified for the GMM network resulting in 7230 transcripts connected by 14908 edges (Supplemental Table S1). The GMM network is presented in Fig. 2. The network demonstrates modular, hierarchal and scale-free properties as demonstrated by the linear relationship between the node distribution plot in Fig. 2B (scale-free) and the average clustering coefficient plot in Fig. 2C (modular and hierarchical).

When GMMs are applied to pairwise correlation, distinct modes can be identified. Briefly, the GMM approach attempts to model the pair-wise relationship via a mixture of Gaussian density distributions that can conceptually be visualized as a collection of 3-dimenisonal Gaussian distributions overlaying a 2D scatterplot. Peaks of each distribution form where points are most dense, and the shaped of the distribution conforms to nearby points. The GMM method is Bayesian and the maximum likelihood for distribution parameters (e.g. mean and variance matrices) are updated using a series of successive steps. Unlike many clustering algorithms, only the maximum expected number of clusters need be provided. Through a method known as the Integrated Completed Likelihood (ICL), a prediction is made as to the number of clusters found. Therefore, no *a priori* number of clusters need be provided and the best number of clusters for the data are predicted. This is well suited for a large number of unsupervised pairwise comparisons. Once completed, every sample receives a “label” using the membership probabilities, indicating the distribution to which it most likely belongs.

**Figure 3:**
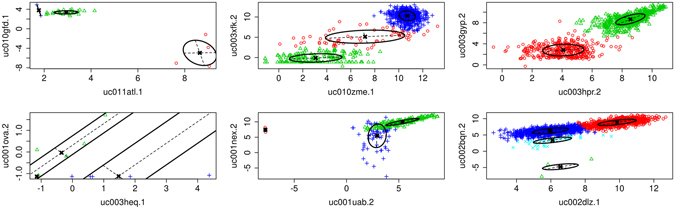
GMM Pairwise Gene Expression Scatterplots. The Gaussian Mixture Model (GMM) algorithm is applied to the same random examples shown in Fig. 3. Each cluster (mode) of samples is identified with a different color. The position and orientation of the Gaussian variance of each cluster is indicated with a black circle and the cluster centers are indicated at the intersection of the variance axis.

Some results of the pairwise application of GMMs can be seen in Fig. 3—the same comparisons from Fig. 1 are provided. Samples are colored according to the distinct modes detected using GMM. These unique modes form “clusters” of samples, and correlation analysis is employed on each cluster individually. Therefore, in the top right plot of Fig. 3, two clusters are identified (a green and red cluster), and each undergo correlation analysis separately. Then, only if a cluster shows correlation will an edge be added to the network. Thus, if multiple clusters were to show high correlation the gene pair could have multiple edges between them in the network. In the case of the top right panel, the green cluster will be more highly correlated whereas the lower red cluster will be lowly correlated. In the case of the middle top plot, neither of the clusters are correlated and no edge will be present in the network for that gene comparison. Next, each edge in the network is annotated with the set of samples that produced it. For example, in Fig. 3, the green cluster in the top right panel will yield an edge in the network. That edge will be annotated such that each of the samples colored green will be associated with that edge. The bottom right panel seems to indicate that both the red and blue clusters could be two separate edges in the network (as each appear correlated). Despite that the two edges would occur between the same two transcripts, each would be annotated separately. In practice, the annotation is made using a string of digits where 1 indicates presence of the sample in the cluster and 0 indicates absence. Samples that had no expression measurements are assigned a 9 and outliers are assigned a 6 or 8 depending on the outlier removal step. Each edge, therefore, has a “sample string” consisting of 2016 digits (see example sample strings in Supplemental Table S1).

After the GMM network was constructed, modules were identified using the Link Communities method (LCM)^56, 57^. The LCM method was selected because it allows a gene to be present in multiple modules which supports the understanding that some genes are pleiotropic. A total of 356 modules were identified in the GMM network (Supplemental Table S2). Samples that are present in 95% of the edges in a module were used to test for significant enrichment of cancer subtype (LGG, THCA, GBM, OV, and BLCA) as well as other clinical annotations provided by TCGA including gender, cancer stage, and ethnicity. The full results of this enrichment analysis are provided in Supplemental Table S3. Table 1 provides a summary of the number of modules with significant annotations (Fisher’s Test *p* < 0.001). Additionally, functional enrichment analysis was performed for each module and provided in Supplemental Table S6. A selection of interesting modules with enriched clinical and molecular annotation is explored in the Discussion section. It is important to note that some modules in Table 1 may be enriched for multiple types of cancer, and these relationships can be seen in Supplemental Table S3.

**Table 1:** GMM Network Modules with Enriched Clinical Annotations.

**Cancer Types**						
BLCA	OV	LGG	THCA	GBM		
13	15	32	9	18		
**Gender**						
Female	Male					
11	22					
**Cancer Stage**						
Stage I	Stage II	Stage III	Stage IV	Stage IVA	Stage IVC	
10	3	0	10	5	0	
**Ethnicity** ^*^						
NHL	HL	W	AA	A	NHPI	AIAN
2	3	22	0	6	0	0

Each value indicates the number of modules in the GMM network enriched for the specified annotation with a *p*-value < 0.001. BLCA (bladder cancer), OV (ovarian cancer), LGG (lower grade glioma), THCA (thyroid cancer), GBM (glioblastoma), NHL (not Hispanic or Latino), HL (Hispanic or Latino), W (White), AA (African American), A (Asian), NHPI (Native Hawaiian or Pacific Islander), AIAN (American Indian, Alaska Native).

### Condition Specific Sub Networks

**Figure 4:**
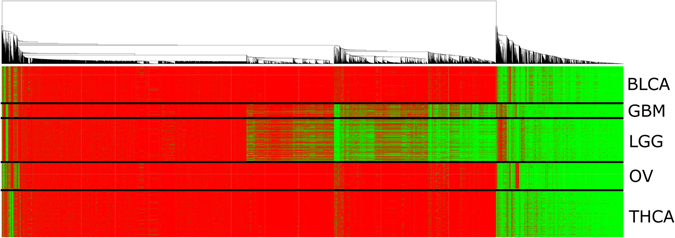
Network Sample Composition Heat map. Each edge in the network is annotated with a string of 1’s and 0’s, referred to as a sample string. For the human cancer network, each string consists of 2016 1’s and 0’s with a 1 indicating that the sample is present within the cluster that formed the edge, and a 0 indicating it is not included. Hierarchical clustering using the heat map function of the R statistical package was used to order edges by similarity of their sample strings and generate this figure. Here, red indicates the presence of a 0 in the sample string and green indicates the presence of a 1. Each column of the heat map represents an individual edge in the network. Samples represent rows in the heat map and are grouped according to cancer types (i.e. BLCA, GBM, LGG, OV and THCA). An artificial black line was added to distinguish between “lanes” of each cancer type.

To visualize condition-specific edges in the GMM network, a heatmap can be generated using the sample strings for each edge. To this end, sample strings are converted to a numeric matrix. In practice, any digit other than a one is converted to a zero, then the matrix undergoes hierarchical clustering and finally plotted as a heatmap. Thus, edges that are most similar in terms of sample composition are re-ordered near each other. Figure 4 shows a heat map with 1’s displayed in green and 0’s displayed in red. Each column of the heat map represents a single edge in the network (i.e.14908 columns). Each row represents a sample (i.e. 2016 rows). Samples are grouped by cancer type in the ordering of the rows and differentiated using black horizontal lines.

Upon inspection, the rightmost portion of the figure shows the components of the network that consists of relationships common across all cancers. This is indicated with near solid green across all cancer lanes. Moving from the far right to the left, the edges begin to show cancer-specific co-expression. For example, both brain cancers (GBM and LGG) show many vertical streaks of green when other cancer types show red. These edges therefore demonstrate co-expression specific to brain cancer only. Other cancers such as bladder, ovarian and thyroid have much smaller representation in the network. Edges that are uniquely part of these tumor subtype are evident by the smaller stripes of green at the far-left side of the heatmap. Interestingly, there is a red vertical strip in the ovarian cancer lane (OV) near the right of the figure. Other cancer types show green within those edges. This indicates co-expression that is not present in ovarian tumors yet is present in other tumors. Additionally, many edges in the network have no relationship with a cancer type. These are indicated by the large section of primarily red samples in the left side of the heat map.

### Comparison of GMM versus Non-GMM Networks

**Figure 5:**
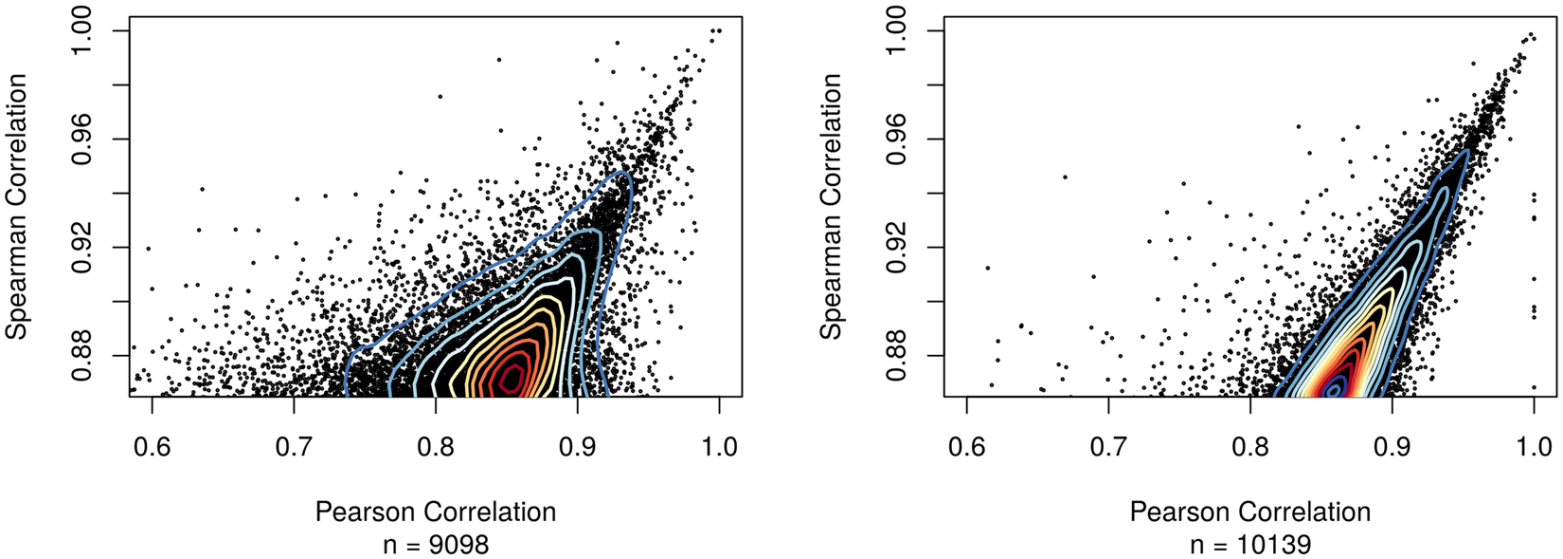
Non-GMM vs GMM PCC and SCC values. (**A**) The scatterplot of Pearson vs Spearman correlation from the edges of a cancer network constructed using the same input dataset, but created without usage of GMMs. (**B**) A similar plot but from the GMM network. The number of points in both panels (corresponding to edges in the network) is indicated by the variable *n*. Contour lines have been added to indicate point density.

As mentioned previously, correlation methods can erroneously introduce edges into the network due to co-modality rather than co-expression. This occurs because multi-modality in a comparison breaks the assumptions of Pearson—the sample variation is not homoscedastic. Spearman correlation does not require homoscedasticity but it cannot distinguish when modes are present. Use of GMMs, however, allow correlation methods to be applied more appropriately because each mode underlies a set of samples following a Gaussian distribution which is inherently more appropriate for the assumptions of Pearson and allows Spearman to focus on each mode separately. Spearman and Pearson often exhibit a range in coefficients from the same set of comparisons. For example, Pearson and Spearman-based network were created without GMMs using the same cancer dataset. In summary, 3532 edges and 2369 transcripts are in common between the Pearson and Spearman networks (Pearson contains 20183 edges and 3662 transcripts; Spearman contains 14774 edges and 5025 nodes). Thus, a change in correlation statistic can yield a widely different network from the same input data set. Of note, in comparison with the GMM network 1528 edges and 1786 transcripts are in common with the Pearson network and 6014 edges and 3233 transcripts are in common with the Spearman network. Figure 5 indicates that less variation exists between PCC and SCC values after GMMs are employed. Non-GMM Pearson and Spearman networks are available as Supplemental Tables S4 and S4 respectively.

To demonstrate the functional connectivity, or performance, of non-GMM and GMM networks, we used the Extending ‘Guilt-by-Association’ by Degree (EGAD)^58^ software package. The EGAD software calculates functional properties of networks using the guilt-by-association property by measuring how well known genes are grouped together. The average area under the receiver operator characteristic curve (AUROC) using the neighbor-voting algorithm was 0.604 for the GMM network, 0.630 for the Pearson and 0.679 for the Spearman network. An AUROC score of 0.5 is considered random, 0.7 is considered good, 0.9 is considered high performance. To compare these three networks with other published networks we obtained a published bladder (InCaNet BLCA) and ovarian cancer (InCaNet OV) co-expression networks^59^ as well as a protein-protein interaction network that accompanies EGAD (BioGrid network). These networks yield AUROC scores of 0.566, 0.559 and 0.671 respectively. Thus, these performance scores seem to indicate similar functional performance for our non-GMM and GMM networks, but with functional performance better that other published cancer networks (see Supplemental Table S6 for full results).

## Discussion

Some pairwise gene expression relationships exhibit modality. Our hypothesis is that these multi-modal comparisons represent condition-specific gene expression and therefore each mode should be accounted for separately during network construction. GMMs offer a straight-forward approach to identify these modes. The hypothesis is supported in that samples in some groups of edges in the network are clearly differentiated by cancer type (Fig. 4). A natural follow-up question is to ask if these condition-specific edges are connected within the network? As shown in Table 1 and Supplemental Table S3, enrichment of sample annotations was performed on modules detected in the network. Some modules do show strongly significant enrichment for clinical traits. Because modules tend to be more highly connected internally than externally it indicates that some of these co-expressed condition-specific edges can be used for guilt-by-association inferences.

In comparison to the two non-GMM networks (using traditional Pearson and Spearman), it is interesting that the GMM network shares fewer nodes and edges than the two non-GMM networks share with each other. Comparisons that meet the assumptions of the Pearson test (linear, no strong outliers, and homoscedastic across all samples) with high correlation would certainly be captured by the Spearman test and would always be suitable for GMMs—as the samples in a GMM cluster will always meet Pearson’s assumptions after outlier removal. Therefore, the fact that we see so few shared edges between the Pearson and GMM networks indicates that a clear majority of comparisons do not meet Pearson assumptions. In fact, removal of all edges not shared with the GMM network yields a Pearson network (of 1786 nodes and 3084 edges) which is scale free and hierarchical and therefore consists of only linear co-expression relationships. We suggest that Pearson’s should not be used for network construction unless assumptions are checked at each comparison and then only if linear relationships are desired. The GMM network used Spearman for correlation analysis. But, the non-GMM Spearman network is different from the GMM network. The GMM network contains 2205 more transcripts but a similar number of edges with 40% shared edges and 64% shared nodes in the Spearman network. We expect that the discrepancies between these two networks are therefore due to multi-modal comparisons that are inappropriately added in the non-GMM Spearman network, and bias in the GMM approach that yields false edges. The quantity and type of bias created by GMMs is not yet known and requires further investigation. Despite any noise from the GMM approach, condition-specific edges can be identified in the network despite extrinsic noise that accumulates in large disparate data sets.

To demonstrate the potential value of modules identified from the GMM tumor network we examined two GMM modules: M0057 and M0282. The module M0057 demonstrates GMM’s ability to find modules specific for a single tumor subtype as M0057 is enriched for thyroid tumors (p = 6.95 × 10^−152^) but is not enriched for other tumor groups (p = 1.00). The six unique transcripts of M0057 are thyroid genes that are annotated for different types of thyroid peroxidase (TPO), an enzyme expressed primarily in the thyroid. TPO oxidizes anionic iodide into iodine atoms. Tyrosine residues on thyroglobulin are thereafter iodinated during thyroid hormone synthesis^60^. M0057 is also enriched for “female” (p = 2.81 × 10^−6^), Stage I cancer (p = 1.07 × 10^–21^) and the vital status “alive” (p = 7.07 × 10^–36^). Thus, the transcripts in this module may be more relevant to early stage thyroid cancer and possibly more relevant to female patients.

The second module, M0282, is enriched for a broader set of more aggressive tumor subtypes and contains genes involved in cell proliferation. M0282 includes 82 unique transcripts, 839 unique edges, and 1426 of the 2016 samples in the network. This module is enriched for bladder cancer (p = 1.89 × 10^–30^), ovarian cancer (p = 1.81 × 10^–27^), and glioblastoma (p = 2.26 × 10^–11^), but not thyroid cancer (p = 1.00) or lower grade glioma (p = 1.00). M0282 is also enriched for Stage IV cancer (p = 1.20 × 10^–15^) and the vital status “dead” (p = 1.51 × 10^–47^), indicating that this module may be relevant to more advanced tumors than M0057. M0282 is enriched for 82 Gene Ontology (GO)^61^, 48 Reactome^62^, 14 Interpro^63^, 10 PFAM^64^, five KEGG^65^, and four MIM^66^ annotations. Together, these annotations represent a broad spectrum of cell cycle-related activities. For example, the most highly-enriched KEGG and Reactome annotations are “cell cycle” (hsa04110, R-HSA-1640170), and the second-most enriched Reactome annotation is “cell cycle, mitotic” (R-HSA-69278). Furthermore, the 26 most-enriched GO terms mention either “cell cycle,” “mitosis,” “cytokinesis,” “microtubules,” “kinetochore,” “kinesin,” and/or a specific phase of the cell cycle. Indeed, 52 of the 82 GO terms mention these phrases and nearly all the other terms are readily tied to the cell cycle. Also of interest are the Reactome annotations because each phase of the cell cycle is enriched for this module. M phase (R-HSA-68886) is the most-enriched of any phase, but three of the top-18 most-enriched Reactome annotations (R-HSA-453279, R-HSA-69206, R-HSA-69205) mention the G1/S checkpoint, the cell cycle’s point of no return. Fitting with the G1/S checkpoint, p53 signaling (hsa04115) is also enriched for this module. These and other tumor-specific modules detected with the GMM-GCN approach are worthy of further investigation.

While use of GMMs shows promise for identifying condition-specific co-expression, there are challenges. First, the time required to compute a GMM network is greater than constructing a network in a traditional manner. This increase in computational complexity is due to calculating GMMs at each pairwise calculation. The tumor network reported here required one calendar month to complete using the Open Science Grid^67^. Second, the mixture model software library (mixmod)^68^ integrated into KINC uses a random initialization strategy. Therefore, it is possible to identify different clusters depending on the random initialization point. For clearly defined clusters this seems to be a rare problem, but it can result in some clusters being indistinguishable when they are less distinct (i.e. very close and overlapping). Third, GMMs may not be appropriate for every relationship between pairs of genes. For example, imagine a non-linear relationship with low variance that spans from the bottom left to the top right of a scatterplot. In this case, multiple modes along the length of the plot are identified. The resulting clusters are near each other, often have no distinguishable gap between them and hence could be merged into a larger non-linear cluster. Strategies exist for merging GMM clusters^69^ but these are not yet incorporated into KINC.

In summary, we show that modes within pairwise gene expression comparisons can be the result of condition-specific variation (extrinsic noise). GMMs are a practical approach to reduce this noise via segregating datasets into modes prior to pairwise correlation analysis. Thus, a GCN can be constructed from mixed condition sample sets where conditions are parsed in a knowledge independent manner. Since samples in a mode are captured as strings and included as edge annotations, one can associate sample meta data (e.g. tumor sub-type, clinical traits) and molecular information (e.g. pathways) to individual edges and edge communities (modules) to reveal higher order associations between gene output and traits.

## Methods

### Implementation of Gaussian Mixture Models

The Knowledge Independent Network Construction (KINC) open-source package is the successor to RMTGeneNet^39, 54^. RMTGeneNet was rebranded to convey the idea that the employed methods used for network construction proceed in a knowledge independent fashion. Stated differently, no *a priori* knowledge about the structure of the network is used to bias construction. KINC provides knowledge independent construction through use of correlation analysis (Spearman, Pearson and MI), network thresholding using Random Matrix Theory (RMT)^55^, and the newly added support for GMMs which identify subpopulations of samples for each pairwise gene comparison. Support for clustering using GMMs was integrated into KINC v1.0 using the C +  + library mixmodLib^68, 70^. The mixmodLib package supports several criteria for model selection including Bayesian Information Criterion (BIC), the Integrated Completed Likelihood (ICL), the Normalized Entropy Criterion (NEC) and a few others. ICL performed best for correlation of pairwise gene expression and was used for all clustering. The mixmodLib package also supports a variety of Gaussian models that vary in volume, shape and orientation. KINC currently supports the default model family as provided by the mixmodLib package.

### Overview of Gaussian Mixture Models

Finite mixture models are probabilistic models, or a mixture distribution, used for density estimation, identification of subpopulations and discriminant analysis. For this work, we use Gaussian mixture models for identification of subpopulation (i.e. modes). Here we provide a brief introduction to GMMs but more in-depth descriptions can be found within a large collection of literature.

Let **x** = {**x**_1_, …, **x**_*n*_} be the set of data vectors to be considered. For this study *n = *2 because we compare two genes at each comparison and each vector is an expression profile of a gene. Each **x**_*i*_ arises from a probability distribution estimated by the function.

$$f({x}_{i}|\theta )=\sum _{k=1}^{K}{p}_{k}h({x}_{i}|{\lambda }_{k})$$

where *K* is the number of components (i.e. subpopulations), *h*(·| λ_k_) is a *d*-dimensional density distribution function parametrized by λ_k_ (in this case *d* = 2 because we are comparing two genes), where λ = (μ, Σ) and μ is the mean of the distribution with covariance matrix Σ. Because *d = *2 for this study the variance matrix is a 2 × 2 matrix. The parameter, *p*_*k*_ is the mixing proportion for each respective component and varies 0 > *p*_*k*_ < 1. Finally, *θ* = (*p*_1_, …, *p*_k_, λ_1_, …, λ_k_) and represents the set of mixing proportions together with the *K* density distributions modeled by their means, (μ_1_, …, μ_k_), and covariance matrices, (Σ_1_, …, Σ_k_), that are to be estimated.

To estimate *θ* the expectation-maximization (EM) algorithm^71^ is used. It is an iterative method that finds the maximum likelihood for the parameters. To begin, a randomization initialization step is performed. At this step, the mixing proportions *p*_*k*_ is initialized to 1/*n* (where *n* = 2), and *θ* ^0^ is initialized such that each mean, is randomly assigned an existing sample coordinate. The mixmodLib package supports a variety of Gaussian families that supports spherical or elliptical distributions with variation in volume, orientation and shape. For this study, the default family was used (called the Gaussian_pk_Lk_C family and described as an “Ellipsoidal Gaussian model with free proportions”). The EM algorithm then consists of two repeating steps: an E-Step and a M-Step. The E-step calculates the current conditional probabilities that any point, ***x***_*i*_ belongs to a distribution *k* using the current *θ* values. For brevity, formulas for the E and M steps are not shown—readers are referred to available literature for further details. The M-Step then updates *p*_*k*_ and *θ* by calculating the maximum likelihood using the conditional probabilities. The EM algorithm continues for *m* number of trials, where *m* is set by the user, with the updated *p*_*k*_ and *θ* used in subsequent steps. Additionally, the mixmod package provides alternatives to the EM algorithm including a stochastic EM method, SEM, and a method that includes a classification step between the E and M steps knows as CEM. For this study we used the default EM approach.

A major advantage of the mixmodLib package is that it can select a model and the number *K* such that users need not provide *K a priori*. User’s simply provide an upper limit for *K*. There are several model selection algorithms provided by the mixmodLib package including the Bayesian Information Criterion (BIC), the Integrated Completed Likelihood (ICL) and the Normalized Entropy Criterion (NEC). Each performs best under different applications. BIC is best suited for density estimation applications, ICL and NEC are best for subpopulation identification but NEC is meant simply for choosing *K* rather than *K* and *θ*. Therefore, for this study we used the ICL method to determine the number of subpopulations at each pairwise comparison. Readers are referred to the literature for more in-depth descriptions of these model-selection techniques. More in-depth mathematic descriptions for these methods are provided in the statistical documentation on the mixmod website.

### TCGA GEM Construction

All normalized isoform datasets for lower grade glioma (LGG), thyroid cancer (THCA), glioblastoma (GBM), ovarian cancer (OV), and bladder cancer (BLCA) were downloaded from The Cancer Genome Atlas^49^ on April 1 2016. In total, 2016 Level-3 RNA-Seq datasets (RPKM units) were downloaded that comprised of 534 LGG, 572 THCA, 174 GBM, 309 OV, and 427 BLCA samples. These expression values were created with TCGA’s RNASeqV2 workflow^72, 73^, which uses MapSplice to map reads and RSEM to quantify mapped reads. All 2016 expression vectors containing 73599 transcript (UCSC kg5 identifiers) quantifications were combined into a single Gene Expression Matrix (GEM). In this GEM, all missing values were replaced with the word, ‘NA’, as is usually indicative of missing values in the R statistical package. A log2 transformation of the expression values was performed, followed by a Kolmogorov-Smirnov test using the preprocessCore^74^ R library to test for samples demonstrating abnormal density distributions (D_N_ > 0.15). No significant outliers were detected. Finally, quantile normalization was performed, also using the preprocessCore library, to ensure suitable comparison between samples.

### GMM Network Construction

Due to the high amount of computational time required for pairwise GMM (2.9 billion comparisons = (*n* x (*n* − 1))/2, where *n = *76300 and is the number of transcripts in the TCGA GEM), the Pegasus Workflow Management System^75^ was utilized to direct the execution of 75,000 jobs on the Open Science Grid (OSG). Spearman correlation was used for each cluster identified by the GMM method. Only clusters with 30 or more TCGA samples underwent Spearman correlation. Thirty samples were identified as the minimum number using a Pearson’s power analysis to ensure a false positive rate at α = 0.05, a false negative at β = 0.2 and an effect size of 0.5. Once completed, the resulting files that represent the correlation matrix were transferred to either Clemson University’s Palmetto Cluster using Globus^76^ or Washington State University’s Kamiak cluster for identification of a correlation threshold using Random Matrix Thresholding (RMT). During thresholding, clusters with fewer than 30 samples or those with extremely low range of expression (less than 0.1) were ignored. KINC v1.0 outputs the correlation matrix as a set of correlation files. Entries from the result files with perfect correlation (*ρ* = 1.0) were removed as there were approximately 360000 of these relationships. These perfect correlations appear to be the result of low range of expression. KINC generates a network file containing edges and metadata that represent the network.

### Non-GMM Network Construction

Two additional networks were constructed using KINC v1.0 with this same 2016 × 73599 TCGA GEM but without use of GMMs. Rather, traditional use of both Spearman and Pearson correlation was employed. These networks were constructed using a single CPU each on Clemson’s Palmetto cluster.

### Module Detection and Analysis

Subgraphs of highly interconnected nodes (i.e. modules) were identified in the GMM-based cancer network using the linkcomm R package^56, 57^. The linkcomm package employs the Link Communities approach for module detection and clusters “links”, or edges, rather than nodes. This allows nodes (i.e. gene transcripts) to be present in multiple modules. This method was selected for clustering under the assumption that some genes are pleiotropic and hence present in more than one biological function. Functional enrichment of these modules was performed using an in-house Perl script modeled after the online DAVID tool^77^ but is fully command-line integrated and supports any number of functional input files. Functional lists used for functional enrichment include human transcripts mapped to terms from InterPro^63^, PFAM^64^, the Gene Ontology (GO)^61^, the Kyoto Encyclopedia of Genes and Genomes (KEGG)^65^, Reactome^62^ and MIM^66^. Terms that were present in a module more often than in the genomic background were considered enriched (Fisher’s test; *p* < 0.001). Results from this functional enrichment analysis are found in Supplemental Table S7 and include *p*-value corrected for multiple tests using both Bonferroni and Benjamini.

### Module Enrichment of Clinical Annotation

In addition to edge lists, KINC v1.0 output includes metadata such as sample list strings that were used to identify each significant edge. These samples, in this case tumors, represent the GMM cluster and the presence of the edge indicates correlation of gene expression across these samples. Additionally, each sample obtained from TGCA has a variety of clinical annotations including cancer type, ethnicity, and gender. To test if a module was enriched with a particular annotation, a Fisher’s Exact Test was performed to identify any cancer annotations that are significantly more present than would be expected from a random selection of samples (*p* < 0.001). For stringency, only samples that are present in 95% of the edges of the module are counted. Not all of the samples had values for all of the annotation types, therefore, we selected those that were annotated across a broad set of samples. These include cancer type, gender, cancer stage, and ethnicity.

### Functional Performance Analysis

The guilt-by-association performance of the GMM network was compared to the non-GMM Pearson and Spearman networks using the Extending ‘Guilt-By-Association’ by Degree (EGAD) software^58^. Two public cancer lnCaNet co-expression networks that were constructed using TCGA data (‘lncanet_BLCA_Cancer’ and ‘lncanet_OV_Cancer’), were downloaded for comparison^59^. In addition, protein-protein interaction data from BioGrid that is included in the EGAD R package was also used. Gene Ontology (GO) terms mapped to TCGA transcript ID’s were used as the annotation vector for the GMM, and non-GMM Pearson and Spearman networks. For the lnCaNet networks and the BioGrid data, the GO term annotation vector provided in the EGAD R package was used to extract the appropriate labels (common gene name or Entrez ID) mapped to GO term IDs. All input gene sets were treated as sparse, binary networks.

### Data Availability

All data is provided as Supplemental Figures, Tables are made available with this article.

## Electronic supplementary material

Supplemental Figure

Supplemental Tables
