# Supplementary material for: Discovering Condition-Specific Gene Co-Expression Patterns Using Gaussian Mixture Models: A Cancer Case Study
Source: Sci Rep. 2017 Aug 17;7:8617. doi: 10.1038/s41598-017-09094-4 (PMC5561081; doi:10.1038/s41598-017-09094-4)
Supplement: Supplementary file 1 — Supplemental Figure [file 41598_2017_9094_MOESM1_ESM.doc]

**Discovering Condition-Specific Gene Co-Expression Patterns Using Gaussian Mixture Models: A Cancer Case Study**

Stephen P. Ficklin^1^*, Leland J. Dunwoodie^2^, William L. Poehlman^2^, Christopher Watson^3^, Kimberly E. Roche^2^, F. Alex Feltus^2^

^1^Department of Horticulture, Washington State University, Pullman, WA 99164, USA

^2^Department of Genetics & Biochemistry, Clemson University, Clemson, SC 29631, USA

^3^Molecular Plant Sciences Program, Washington State University, Pullman, WA 99164, USA

***Corresponding Author Contact Information**

Stephen Ficklin, PhD

Assistant Professor

Bioinformatics and Systems Genetics

Department of Horticulture

Washington State University

153 Johnson Hall

Pullman, WA 99164-6414

Office: (509) 335-4295


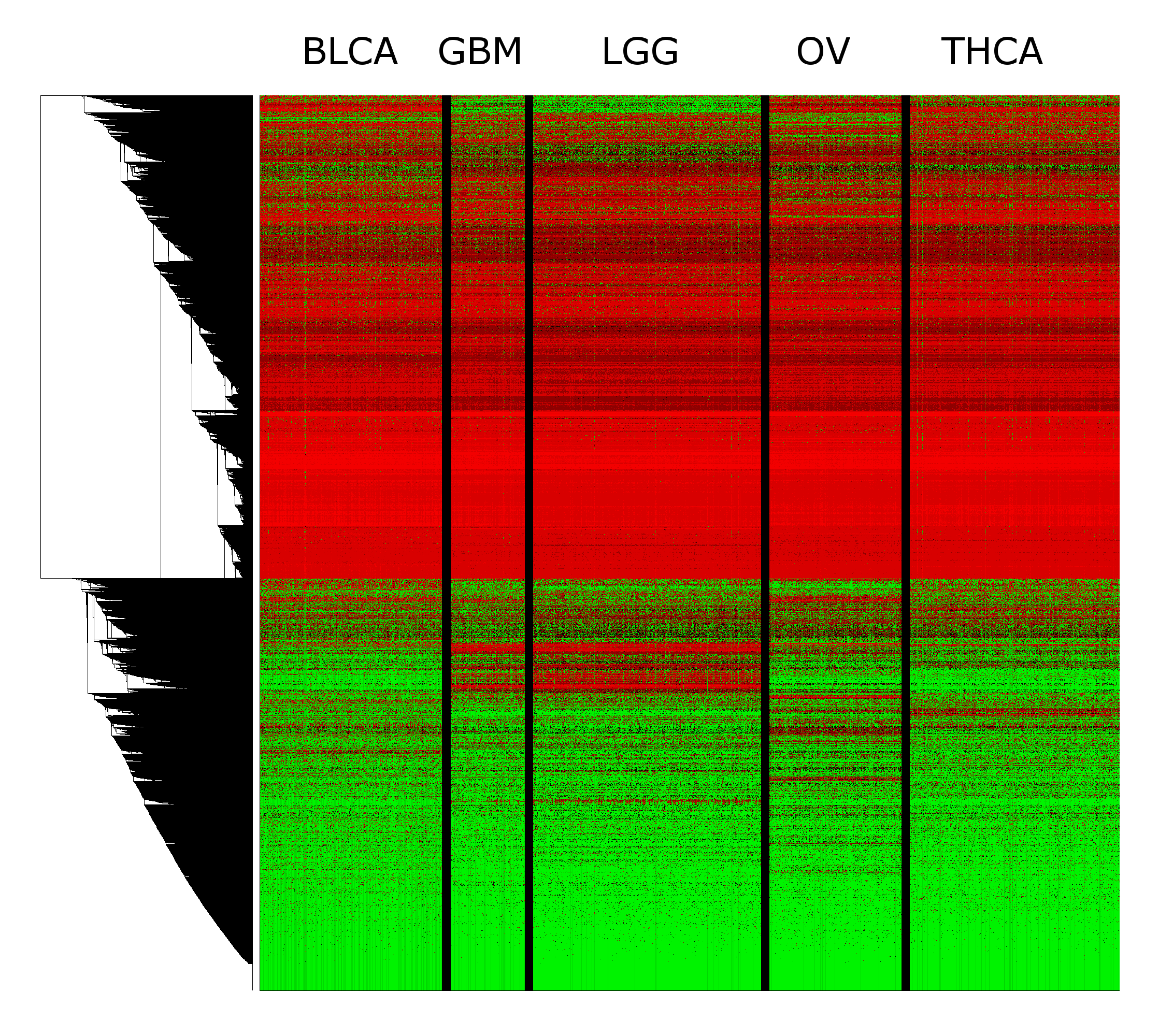


**Supplemental Figure S1.  Global Tumor Gene Expression.**For this heat map, rows are transcripts and columns are samples. Sample are grouped by bladder cancer (BLCA), glioblastoma multiforme (GBM), low grade glioma (LGG), ovarian cancer (OV), and thyroid carcinoma (THCA)*.* Red indicates higher gene expression while green indicates lower expression. Transcripts were clustered on the y-axis using the clustergram Matlab function.
